# Quantification of Biliverdin, Bilirubin and Bilirubin Glucuronide in Micro-Samples of Animal Blood

**DOI:** 10.3390/bios16100529

**Published:** 2026-09-22

**Authors:** Paola Sist, Federica Tramer, Marco Stebel, Donatella Volpatti, Sabina Passamonti

**Affiliations:** 1 Department of Life Sciences, University of Trieste, Via Giorgieri 1, 34127 Trieste, Italy; psist@units.it (P.S.); ftramer@units.it (F.T.); stebel@units.it (M.S.); 2 Department of Agricultural, Food, Environmental and Animal Sciences, University of Udine, Via Sondrio, 2/A, 33100 Udine, Italy; donatella.volpatti@uniud.it

**Keywords:** HUG fluorometric assay, nanoscale analysis, bilirubin, bilirubin glucuronide, biliverdin, whole blood, vertebrates, *Rattus norvegicus*, *Dicentrarchus labrax*, *Sparus aurata*

## Abstract

Circulating bile pigments are routinely measured in human and veterinary medicine, toxicology, pharmacology, and biomedical studies, as they are consolidated biomarkers of liver dysfunction and hemolysis and emerging biomarkers of biological responses to stress. This study was performed to extend bile pigment analysis from plasma to microvolumes of whole blood not only to simplify the pre-analytical steps but also to surmount the bottleneck of limited blood volume available for withdrawals in screening and monitoring. We applied the HUG assay for the nanoscale analysis of biliverdin, bilirubin, and bilirubin glucuronide. HUG is a chimeric protein expressing the UnaG domain that emits fluorescence upon binding bilirubin. This study shows that, under specific conditions, the HUG assay enables quantification of the three bile pigments in rat and fish whole blood. Moreover, quantification is equivalent in blood, plasma and serum. This approach skips pre-analytical steps, reduces errors and time to analysis, thereby facilitating research of circulating bile pigments in *animalia* while preserving the possibility of comparing new data with historical sets of clinical chemistry data in the literature.

## 1. Introduction

Bile pigments comprise three open tetrapyrrolic compounds, i.e., biliverdin (BV), bilirubin (BR) and bilirubin glucuronide (BRG). They account for the color of the bile, into which they are excreted. Derived from heme cleavage by the action of heme oxygenase (E.C. 1.14.99.3) [1], BV is reduced by biliverdin reductase (BVR, E.C. 1.3.1.24) to BR. These bile pigments are found in the blood and give plasma/serum its color, which may appear either pale yellow, as typically in humans, because of the presence of BR, or blue green, as in some fishes, because of BV [2]. Both BV and BR are taken up into the liver and excreted either unchanged or as BRG into the bile [3]. In human and veterinary medicine, plasma BR and BRG are consolidated biomarkers of hemolysis and a spectrum of liver dysfunctions, consisting of defective uptake or glucurono-conjugation and biliary excretion [4]. As such, they are monitored not only in clinical settings, but also in preclinical studies using model rodents [5]. In toxicology, they are mandatorily measured because both erythrocytes and the liver may be primary targets of drugs and toxicants [6]. In some fish species, an increase in the BV/BR ratio may indicate exposure to environmental stress and starvation [7,8], providing indications on animal’s adaptation to the environment. The comparative study of bile pigments in animal blood remains poorly explored, although such knowledge is necessary both to acquire historical data of clinical chemistry from laboratory animals [9] and to gain insights in how *animalia*, whether farmed or wild, regulate their catabolism of heme and the pathway of excretion of biliary pigments in health and disease. It must be reminded that heme oxygenase (HO) is encoded by two genes, of which HO-1 is tightly regulated at the transcriptional level by several endogenous metabolites and environmental stressors [10]. Conventional methods to measure BR in the plasma or serum are automated and based on colorimetric reactions that provide total bilirubinemia (Tbil, i.e., bilirubin and bilirubin glucuronide) or fractionated bilirubinemia (BR and BRG, also termed indirect bilirubin, Ibil, and direct bilirubin, Dbil, respectively) [11]. More advanced methods are based on HPLC [11], some of which enable the simultaneous quantification of BR and BV [12], although they are based on advanced detection devices. A common feature of the above-mentioned methods is that they require sample preparation steps and, consequently, properly large blood volumes. However, in small animals, the limited blood volume available for analysis can make screening difficult or sometimes impossible.

The purpose of this study was to demonstrate the applicability of the HUG assay in quantifying the three bile pigments in a few µL of diluted whole blood. HUG (acronym of HELP-UnaG) is a chimeric synthetic protein [13], in which the elastin-like domain HELP [14] is fused with the BR-specific fluorophore UnaG [14]. By running parallel tests in the absence or presence of BVR or beta-glucuronidase (E.C. 3.2.1.31), the HUG assay enables to quantify not only BR, but also BV and BRG in the same sample of whole blood. We present below a series of tests that demonstrate the equivalence of measurements of the three bile pigments in the plasma or serum or whole blood of rats and fishes. This body of evidence advances research of blood bile pigments in *animalia* while preserving the possibility to compare new data with historical set of clinical chemistry data in the literature.

## 2. Materials and Methods

### 2.1. Chemicals

Analytical-grade chemicals purchased from Merck KGaA (Darmstadt, Germany), were BR (purity 99%), Bovine Serum Albumin Fraction V (BSA, purity > 98%), BV (purity > 97%), human Biliverdin reductase A (BVR, 1.5 IU/µL), β-Glucuronidase 35 IU/µL), Dulbecco’s Phosphate-Buffered Saline (PBS), NADPH tetrasodium salt in reduced form. Ultrapure milliQ water was used to prepare each solution. HUG was synthesized and purified as described in detail [15]. Black, 96-well microplates (Nunc^®^, purchased by Thermofisher Scientific (Waltham, MA, USA), code 237107; polystyrene, sterile, non-treated surface).

### 2.2. Animals

The methods and results of this study comply with the ARRIVE guideline [16].

#### 2.2.1. Rats

Female Wistar rats (*Rattus norvegicus*, Charles River Italy Inc., Calco, Italy) were housed in GR900 cages (5 animals per cage) (Tecniplast, Buguggiate, Italy) with JELUXYL HW 300/500 (JELU-WERK, Rosenberg, Germany) bedding, under constant environmental conditions (room temperature 21–23 °C, humidity 40–60%, 12 h light/dark cycle, air changes 25 volumes/h) and ad libitum access to VRF 1 (P) feed (SDS) and tap water. Animals (*n* = 20; 220–250 g body weight, b.w.) were anesthetized via intraperitoneal injection of 0.5 mL containing Zoletil (2 mg/kg b.w.) and Xylazine (8 mg/kg b.w.). Monitoring of body temperature with a mercury thermometer placed in the abdominal cavity and respiratory rate were performed every 10 min. Cardiovascular function was monitored by gravimetric measurement of the blood sample volume collected in chronometric time intervals every 5 min.

#### 2.2.2. Fishes

Adult sea bass (*Dicentrarchus labrax*; *n* = 7) and sea bream (*Sparus aurata*; *n* = 7) individuals were hosted in recirculation-based seawater tanks at the experimental facility of the University of Udine at a temperature of 22 ± 2 °C and 8.1 mg/L of O_2_. Prior to sampling, fish were anesthetized with MS222 (Merck KGaA (Darmstadt, Germany) at a dose of 60–150 mg/L according to [17], until they lost responsiveness to visual and tactile stimuli, while maintaining a normal respiratory rate.

### 2.3. Blood Collection

#### 2.3.1. Rat Blood Collection and Serum or Plasma Preparation

When serum was needed, whole blood (ca. 1 mL) was obtained by trans-thoracic cardiac puncture (*n* = 16 rats) and kept at room temperature for one hour. The clot was then separated by centrifugation at 4000 rpm (1377 *g*) for 10 min and the supernatant serum was used for bile pigment analysis. These rats were then euthanized in the home cage using 100% carbon dioxide.

All other whole blood and plasma samples were obtained by repeated withdraws from the isolated femoral artery in individual anesthetized rats who underwent bile duct obstruction to achieve graded increases in BRG in the blood, as described below.

Preparation of the cholestatic rats (*n* = 4) began with isolation of the femoral artery, following a procedure described in detail elsewhere [18]. Briefly, a skin incision was made in the right femoral triangle. The muscles that limit the triangle (*M. sartorius* and *M. abductor longus*) were pulled apart to allow access to the femoral neurovascular bundle. The fascia covering it was carefully incised and the femoral artery was separated from the femoral nerve. A ligature was tightened around the distal end of the artery segment, and a proximal transient occlusion of the artery was achieved by means of pincers. A ligature was prepared at the level of this occlusion but kept provisionally open. Polyurethan tubing (Rat CAC 2 Fr, Instech Laboratories Inc., Plymouth Meeting, PA, USA), was inserted into a small cut in the artery, midway between the distal and proximal ligatures and allowed retrograde sliding inside the artery for 1 cm. Once the proximal ligature had been tightened, 500 U heparin in 0.5 mL phosphate-buffered saline solution (PBS) was injected from an insulin syringe connected to the urethane tubing. The femoral catheter was kept closed by means of pincers.

To obtain blood with varied concentrations of BRG, rat underwent extrahepatic cholestasis by blockade of the bile flux. This was achieved by obstruction of the common bile duct for variable durations (2–20 min). The common bile duct was accessed through a longitudinal laparotomy, allowing it to be isolated. Two ligatures were prepared around the common bile duct about 1 and 2 cm from the hepatic hilus. The distal ligature was tightened, and polyurethan tubing (as above) was gently pushed into the common bile duct and secured by tightening the proximal ligature.

Whole-blood samples (*n* = 20/rat) were collected from the femoral catheter into Eppendorf tubes over 3–5 s (50 ± 6 µL) at different time intervals from the bile duct occlusion. For serum or plasma preparation from these specific cholestatic blood samples, whole-blood aliquots were centrifuged at 4000 rpm (1377 *g*) for 10 min and the supernatant plasma was used for bile pigment analysis.

#### 2.3.2. Fish Blood Collection and Serum/Plasma Preparation

To obtain serum, blood samples were collected via caudal vein puncture using a 2.5 mL sterile hypodermic non-heparinized syringe. Immediately after sampling, a 200 µL aliquot of whole blood was added to HUG solution at a final volume of 2.2 mL. Another aliquot of whole blood (approximately 1 mL) was placed in an Eppendorf tube and allowed to clot, then centrifuged at 4000 rpm (1377 *g*) for 10 min using an Eppendorf centrifuge 5418. The serum aliquots were stored at −80 °C until analysis.

To obtain plasma, blood was collected into heparinized syringes, and plasma was recovered after centrifugation at 4000 rpm (1377 *g*) for 10 min and stored at −80 °C until analysis. After sampling, fish were returned to their original tanks, where they rapidly recovered from the anesthetic treatment.

### 2.4. HUG Fluorometric Assay for the Analysis of Bile Pigments

The assay was performed following the operational steps illustrated in Scheme 1.

#### 2.4.1. Bilirubin Calibration

Aliquots (18 µL) of BSA 4 g/L in PBS, whole blood, serum and plasma were spiked with 2 µL of BR standards in the range 2.5–25 µM (dissolved in BSA 4 g/L in PBS), prepared according to a published protocol that ensures BR stability [19]. After 30 min, samples were diluted 50-fold with 0.05 g/L HUG in PBS to achieve graded BR concentrations in the range 0–50 nM. The microtiter plates were loaded with 200 µL of diluted samples, incubated at 25 °C, and fluorescence intensity (λ_ex_ = 485 nm, λ_em_ = 528 nm; 25 °C) was recorded after 16 h using a benchtop multi-plate reader (Synergy H1; BioTek, Winooski, VT, USA). Matrix-related background fluorescence (samples spiked with BSA 4 g/L in PBS without BR) was subtracted from all other readings.

#### 2.4.2. Bilirubin Spike-and-Recovery Testing

Recovery and matrix effects in rat samples of whole blood, serum and plasma were evaluated following recommendations for untested sample types [20]. Aliquots (45 µL) of each sample type (observed) or 4 g/L BSA solution in PBS, pH 7.4 (expected) were supplemented with 5 µL of BR standards (2, 5 and 10 µM), to achieve low, medium and high increases in endogenous BR levels. For the analysis within the linearity range of the standard HUG assay calibration (0–50 nM), both blood samples (whole blood, serum or plasma) and BSA solution were diluted 20-fold with 0.05 g/L HUG. Each aliquot from 3 independent experiments was analyzed in quadruplicate. BR concentrations were determined directly from fluorescence against the standard curve (mean ± SD, *n* = 3) and recovery was obtained from the net fluorescence of spiked specimens after subtraction of the basal fluorescence.

#### 2.4.3. Rat Samples

Samples (20 µL of heparinized whole blood, plasma or serum) were directly added to the HUG solution (0.05 g·L^−1^ HUG, PBS pH 7.4) to reach a final volume of 2.0 mL. Before their addition to the multi-well plate, diluted whole-blood samples were immediately centrifuged to separate cells. Then, 200 µL of diluted samples was loaded in 8 replicates in black 96-well plates (Nunc^®^) and then either 5 µL PBS, pH 7.4 (for BR analysis) or 5 µL of ß-glucuronidase solution (for BRG analysis) was added to each well (final concentration 0.0875 U/µL). The method was described in detail [21]. A single intrinsic fluorescence value to correct sample fluorescence was obtained by adding a 2 μL rat sample to a final volume of 0.2 mL PBS pH 8.5 and was used to correct sample fluorescence. The microtiter plates were incubated at 25 °C, and fluorescence intensity (λ_ex_ = 485 nm, λ_em_ = 528 nm; 25 °C) was recorded after 16 h using a benchtop multi-plate reader. 

#### 2.4.4. Fish Samples

Aliquots (200 μL) of fish samples (whole blood, plasma or serum) were added to the HUG solution (0.05 g·L^−1^ HUG, PBS pH 8.5) to reach a final volume of 2.2 mL. Before their addition to the multi-well plate, diluted whole blood samples were immediately centrifuged to separate cells. The diluted samples were then divided into three 720 μL aliquots: one for BR, one for BRG, and one for BV quantification. For BR analysis, 80 μL of PBS pH 8.5 was added to one 720 μL aliquot. For BV and BRG, 80 μL of BVR mix (3.7 IU/mL BVR and 1 mM NADPH in PBS pH 8.5) was added to the second aliquot [22], and 80 μL of ß -glucuronidase (final concentration 87.5 IU/mL) was added to the third 720 μL aliquot, respectively. The bile pigment quantification assay was performed in black 96-well plates (Nunc^®^), with each well loaded with 200 μL of sample aliquot. A single intrinsic fluorescence value was obtained by adding 18 μL of fish sample to a final volume of 0.2 mL PBS pH 8.5 and was used to correct sample fluorescence. The microtiter plates were incubated at 25 °C, and fluorescence intensity (λ_ex_ = 485 nm, λ_em_ = 528 nm; 25 °C) was recorded after 16 h using a benchtop multi-plate reader.

#### 2.4.5. Indirect Quantification of BRG and BV

After fluorescence measurement, BR concentration was calculated using the linear regression parameters of the BR calibration curve. The calibration curve obtained with standard BR was used for both bilirubin and total bilirubin derived from the enzymatic reaction of the substrates (BV and BRG), whose completion under the prevailing experimental conditions was previously demonstrated [22,23]. BR concentration in wells supplemented with BVR and NADPH corresponds to the sum BR + BV. From this value, BV was determined by subtracting the BR value (measured in wells without BVR and NADPH). Similarly, the BRG concentration was calculated by subtracting the BR value from the sum of BR + BRG obtained from the wells to which ß-glucuronidase was added.

### 2.5. Hematocrit

Hematocrit measurements in fish blood were carried out in capillary tubes centrifuged with a HEMATOCRIT 4223 Centrifuge for 5 min at 12,000 rpm.

Hematocrit in rat blood was determined by cell counting under an optical microscope. Whole blood was diluted 1:200 in PBS (pH 7.4), and 10 μL of the diluted sample were loaded onto a specialized counting slide with a ruled chamber (Thoma chamber). The average number of red blood cells (RBCs) counted in 16 small squares, with a volume of 0.00025 mm^3^ each, was used to estimate the hematocrit, applying Equation (1):

(1) $$TC\left(\%\right)=RBC\;count\left(\frac{millions}{\mu L}\right)\times MCV\left(fL\right)\times 0.1$$

where HTC is the hematocrit and MCV is the mean volume occupied by red blood cells. The average MCV for the female Wistar rat used was 59.19 ± 2.73 fL, within the reported range of 51.94–63.70 fL [24].

### 2.6. Statistical Analysis

Data were analyzed and plotted using GraphPad Prism version 11.0.2 (GraphPad Software Inc., San Diego, CA, USA). The results are expressed as the mean ± standard deviation (SD), unless otherwise specified.

Calibration curves obtained using PBS-BSA, rat plasma, fish plasma, and rat whole blood as BR solvents were analyzed by linear regression analysis of fluorescence data. The slopes were compared by an extra sum-of-squares F test. Differences between expected and observed concentrations in spike-and-recovery experiments were evaluated using an unpaired Student’s *t*-test. Differences were considered statistically significant when *p* < 0.05.

Correlations between measurements obtained in whole blood, plasma and serum were assessed using Pearson’s correlation coefficient (r) and linear regression analysis.

## 3. Results

We performed this study using either rat (*Rattus norvegicus*) or sea bass (*Dicentrarchus labrax*) and sea bream (*Sparus aurata*) blood. The former is a small-sized mammalian experimental model, and the latter are marine vertebrates of increasing interest for aquaculture and the food market.

A key feature of the HUG assay is that it is calibrated with pure bilirubin prepared in a buffered BSA solution (PBS-BSA) under conditions in which it is stable [19]. The HUG assay of total bilirubinemia in human plasma [21] gives values that are essentially identical to those obtained by the standard colorimetric diazo method [25]. The enzymatic conversion of BV [22] or BRG [23] to BR are performed under controlled conditions, which guarantee that both reactions continue to completion. The BR-HUG complex is stable for at least 72 h [21], ensuring the stability of BR during the assay timeline. HUG can be produced at the laboratory scale according to a detailed protocol that includes quality controls [15].

### 3.1. Analysis of Bilirubin in Whole Blood

The main question we addressed in this study was as follows: Can we measure fractionated bilirubinemia, i.e., the concentrations BR and BRG in whole blood, with the same accuracy as in plasma or serum? The first issue to address was to compare the calibration curves obtained in either the standard solvent (BSA-PBS) or animal plasma and rat whole blood (Figure 1), as per consolidated guidelines [20]. We therefore designed an accuracy test where we added serial BR concentrations (2.5–25 µM) in the standard solvent (4 g/L BSA in PBS pH 7.4, [21]), or in whole blood and plasma collected from fish and rats and then pooled to achieve the working volumes.

There was no statistical difference between the regression coefficients of the standard solution and those obtained with all other matrices, as evaluated by an extra sum-of-squares F test (F(3,70) = 1.536, *p* = 0.2127). These results confirm that the HUG assay measures bilirubin concentrations at the nanoscale without any relevant matrix-dependent interference, thus enabling the use of the BSA solvent for universal signal calibration, irrespective of the types of animal blood or plasma, which may be extremely limiting in some cases. This provides the advantage of directly comparing BR concentrations in the whole blood and plasma of different animals.

### 3.2. Analysis of BR and BRG in the Whole Blood of the Rat

#### 3.2.1. Analysis of BR

Next, we addressed the question of whether BR can be measured in the whole blood of the rat, aiming to skip the step of plasma/serum preparation. As a preliminary step, we verified the absence of interference by heparin with the assay (BR_whole blood_ = 635 ± 20 nM; BR_whole blood + heparin_ = 632 ± 33 nM, values corrected for hematocrit). Next, we conducted spike-and-recovery experiments in rat whole-blood, plasma and serum that were properly diluted as well as in the BR standard solvent, spanning the range 10–50 nM BR. As shown in Table 1, no significant differences were observed between the values measured in the standard BSA solution (labeled as “expected”) and the values observed in the tested samples at any spike level of BR. These results indicate that no matrix effect related to whole blood influenced the assay.

To check the reproducibility and consistency of these findings, we first performed paired analyses of BR in both serum and plasma obtained from several animals. Figure 2A shows a correlation plot of BR measurements in plasma vs. serum (regression coefficient = 0.934 ± 0.101; intercept = 8.05; R^2^ = 0.859). This result enables us to freely choose between plasma and serum samples and to consider them as equivalent in terms of BR measurements. Next, we analyzed the correlation between rat whole-blood and plasma or serum, finding again a strong correlation between the respective quantities (Figure 2B). The slope of the regression curve (0.62 ± 0.03) is an estimate of the fractional plasma volume in this set of whole blood samples, from which the fractional cellular volume could be calculated as equal to 1-slope = 0.38. The latter values can also be expressed in percentage terms, i.e., as hematocrit = 38%. To assess this correspondence, we measured the hematocrit (HTC) in a limited set of blood samples (*n* = 67) by cell counting, obtaining HTC = 42 ± 8%, which is not only similar to the derived value reported above, but also within the reported range and close to the median for Wistar rats (average = 43.27 ± 2.47; range = 37.38–47.89; median = 43.5; [24]). Measurement of the HTC is necessary to apply an experimental correction factor, should whole-blood measurements be compared with plasma/serum measurements, specifically when they are obtained in independent studies.

#### 3.2.2. Analysis of BRG

In medicine, toxicology or experimental medicine, increased blood levels of BRG indicate liver dysfunction. Therefore, it is quite important to monitor it. For this study, pure BRG (either bilirubin mono- or di-glucuronide) would be needed to perform both calibration and spike-and-recovery tests, but it is not available as a commercial standard. Thus, we circumvented this obstacle by designing a plasma vs. blood correlation test at different BRG concentrations, as was done with BR and shown in Figure 2. However, since basal levels of BRG in normal rats are very low, close to the LOQ of this assay and clearly challenging the quality of any correlation, we chose to increase the BRG blood levels by experimental bile duct obstruction, which interrupts the biliary excretion of BRG. By this approach, we could obtain blood samples with BRG concentrations spanning the remarkably wide range 0–1800 nM to ensure the accuracy of the correlation data. The results shown in Figure 3 demonstrate a strong correlation between the values found in either the whole blood or plasma of rats. Figure 3 includes a small set of serum samples (*n* = 7) with detectable [BRG]. The slope of the correlation line was 0.7, reflecting the bias of the blood cell volume.

### 3.3. Analysis of BR, BRG and BV in the Whole Blood of Fish

BV is barely measurable in rat, mouse or human plasma, but it occurs as the main bile pigment in the plasma or serum of some marine fish [22] that have a remarkable impact on the food market in Europe, such as sea bass or sea bream. Monitoring blood bile pigments in farmed fish may support the assessment of fish health and disease, an objective of primary importance not only for producers but also for consumers, environmental and regulatory agencies. The main bottleneck in expanding the panel of blood biomarkers of fish health and wellness results from the combination of the availability of only small blood volumes and the limits of analytical quantification. We previously demonstrated the applicability of the HUG assay for the quantification of BV (and BR) in fish plasma [22]. Here, we assessed the equivalence between measurements in plasma or serum and whole blood (Figure 4) to overcome the inconvenience of collecting blood volumes suitable for effective plasma or serum preparation. The sensitivity of the HUG assay allows significant downsizing, because only 250 µL is needed and can be withdrawn from anesthetized fish specimens (market size of ca. 300 g body weight) that are then returned to the farming pool. In this way, both individualized and collective monitoring can be performed without loss of the fish stock.

Figure 4 shows the correlation of BV, BR, and BRG in plasma vs. whole blood. The data show a strong correlation between the measurements of both BR and BV in either whole blood or plasma. The slope of the correlation line was 0.64 and 0.43, respectively, reflecting the bias of the blood cell volume. A good correlation was also seen when measuring BRG, though data scatter was higher due to the very low values (<14 nM) of BRG in these samples.

However, we observed a systematic underestimation of BR and BRG levels in serum compared to whole blood. The BR concentration in whole blood samples, without hematocrit correction, was 14.3 ± 2.3 nM (*n* = 14), whereas the BR concentration in the corresponding serum samples was 13.2 ± 3.7 nM. The reasons might be ascribed to imprecisions in blood volumes due to the high clotting rate in fish blood and the loss of bile pigments in the process of blood clotting and serum separation, an observation reported for other blood analytes measured in fish species [26]. It is therefore even more important to carry out bile pigment analysis in whole blood rather than in serum to avoid biased results.

## 4. Discussion

The specificity of this study is based on the fact that the quantification of three bile pigments in whole blood was performed not just by conventional spike-and-recovery tests on a single representative matrix specimen, but by comparing measurements of two or three bile pigments in real samples from a number of rats and fishes, respectively.

The rat model of bile duct blockade enabled us to perform the analysis of both BR and BRG in whole blood, despite the lack of commercially available BRG standards for calibration or spike-and-recovery tests. Farmed fish enabled us to perform the analysis of BV, a principal bile pigment in some fish species, but not in rats or humans. Overall, the direct analysis of bile pigments in whole blood, rather than in plasma, provides the opportunity to use very small blood volumes from a single animal and makes repeated withdrawals from the same animal feasible without a major impact on animal physiology. For example, in a rat of 220 g, a blood sample of 50 µL, necessary to measure fractionated bilirubinemia, corresponds to only 0.4% of its blood volume. Since rats are extensively used in toxicology, this method supports the 3R principle in animal research because lower sample volumes enable a higher number of observations, higher power, reduction, and refinement via minimal animal distress.

It must be stressed that this study was not designed to obtain the conventional assay performance parameters using animal whole blood, where a much higher number of measurements is needed to obtain a realistic set of assay performance parameters. While this goal remains to be achieved using species-specific animal blood, we considered that this preliminary study was needed to assess the equivalence of measurements in whole blood versus plasma or serum by correlation analysis. Without evidence of such correlation, the characterization of the performance parameters might be premature because data may not be compared to those existing in the literature and/or current databases of plasma/serum BR concentrations in animals and humans. Our study shows this correlation, even if data may carry yet unsolved uncertainties about the accuracy of measurements. A future goal is the characterization of the HUG assay performance parameters in human blood, where assay accuracy and LOQ are an epistemological requirement to carry out population studies where absolute figures are needed. The same might hold true for population studies in fish species or any other farmed or wildlife animals in ecological surveys, as well as in the case of historical datasets of clinical chemistry parameters in experimental animals.

In future, when applied in human experimental medicine, this assay, which is validated for its performance parameters, would enable the monitoring of blood bile pigments by point-of-care collection and analysis at outposts laboratories. Finally, this method can be applied in ecology studies to investigate emerging biomarkers in aquaculture and aviculture, with a huge impact on human nutrition and related economic sustainability. The equivalence of determinations in the whole blood, plasma or serum of rats and fish enables comparison of new data with a historical set of clinical chemistry data in the literature, thus contributing to the build-up of databases. The plasma concentrations of bile pigments can be calculated from the data obtained from whole blood, provided that the hematocrit is determined. This is absolutely recommended, because the hematocrit may not necessarily be constant, as it depends on the species, as well as on their living and/or experimental conditions. In fish, hematological parameters vary among species [27], or in individual specimens, when contraction of splenic vessels may force stored red cells into the general circulation [28], e.g., due to hypoxia [29], temperature and salinity changes [30], stress [31] and heavy metal intoxication [32].

In conclusion, this work widens the scope of applicability of the HUG assay in monitoring human and animal bile pigments in a microvolume of whole blood, simplifying the step of blood collection and handling. The protocol presented is flexible and several refinements can be done on a case-by-case basis, considering the animal species donating blood and their potential use in clinical and preclinical tests of hemato- or hepatotoxicity of chemicals (e.g., tests of drug or tracer safety) that may interfere with fluorescence emission.

## Figures and Tables

**Scheme 1:**
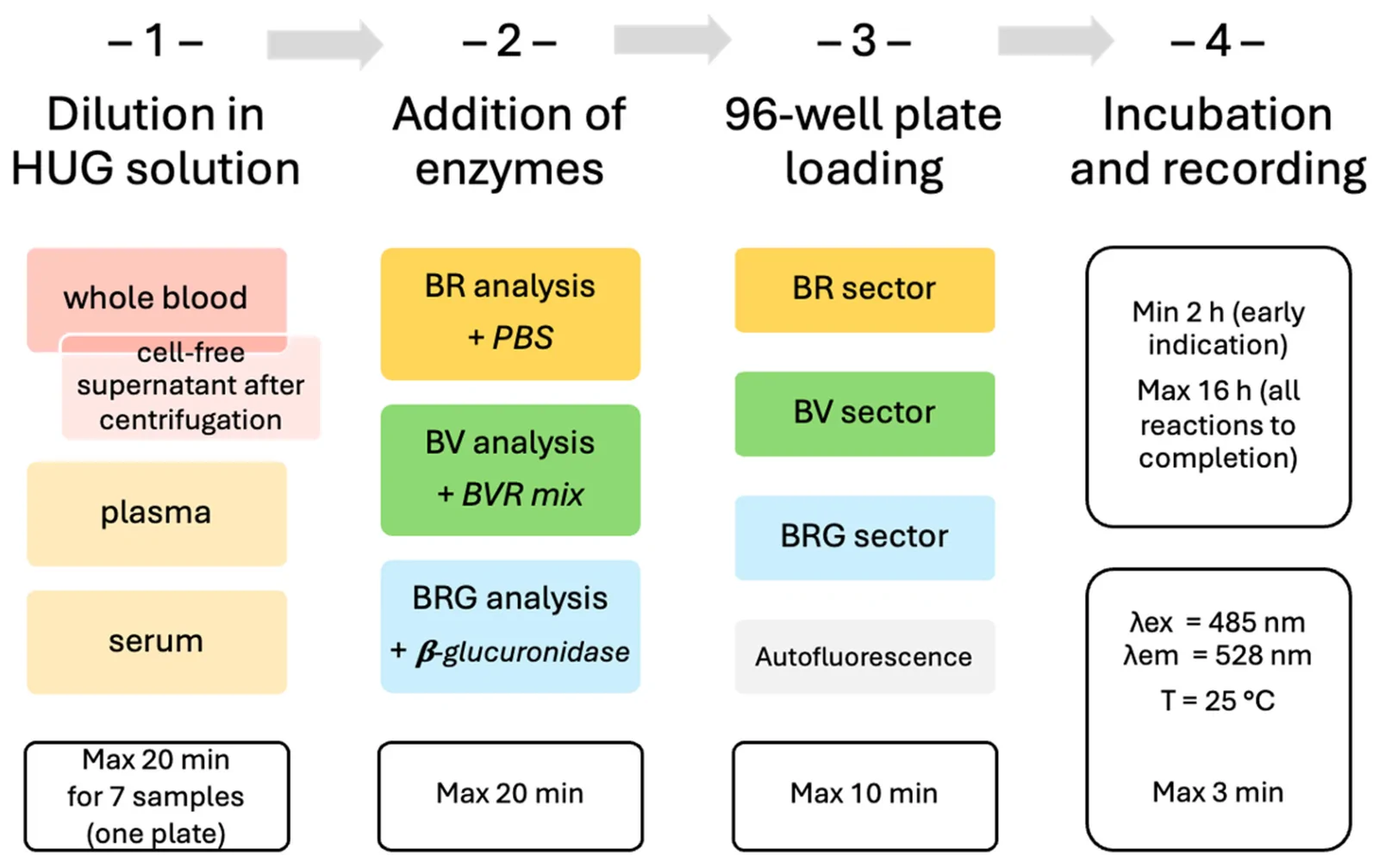
The four main steps of the HUG assay for the combined analysis of bile pigments in animal whole blood, plasma or serum. The cellular fraction of blood is separated after blood dilution in an isotonic solution. Addition of enzymes can be performed either directly in the wells or on separate aliquots of diluted samples before loading onto the plate. Each bile pigment sector contains 7 samples in quadruplicate. The autofluorescence sector contains 1 sample/well.

**Figure 1:**
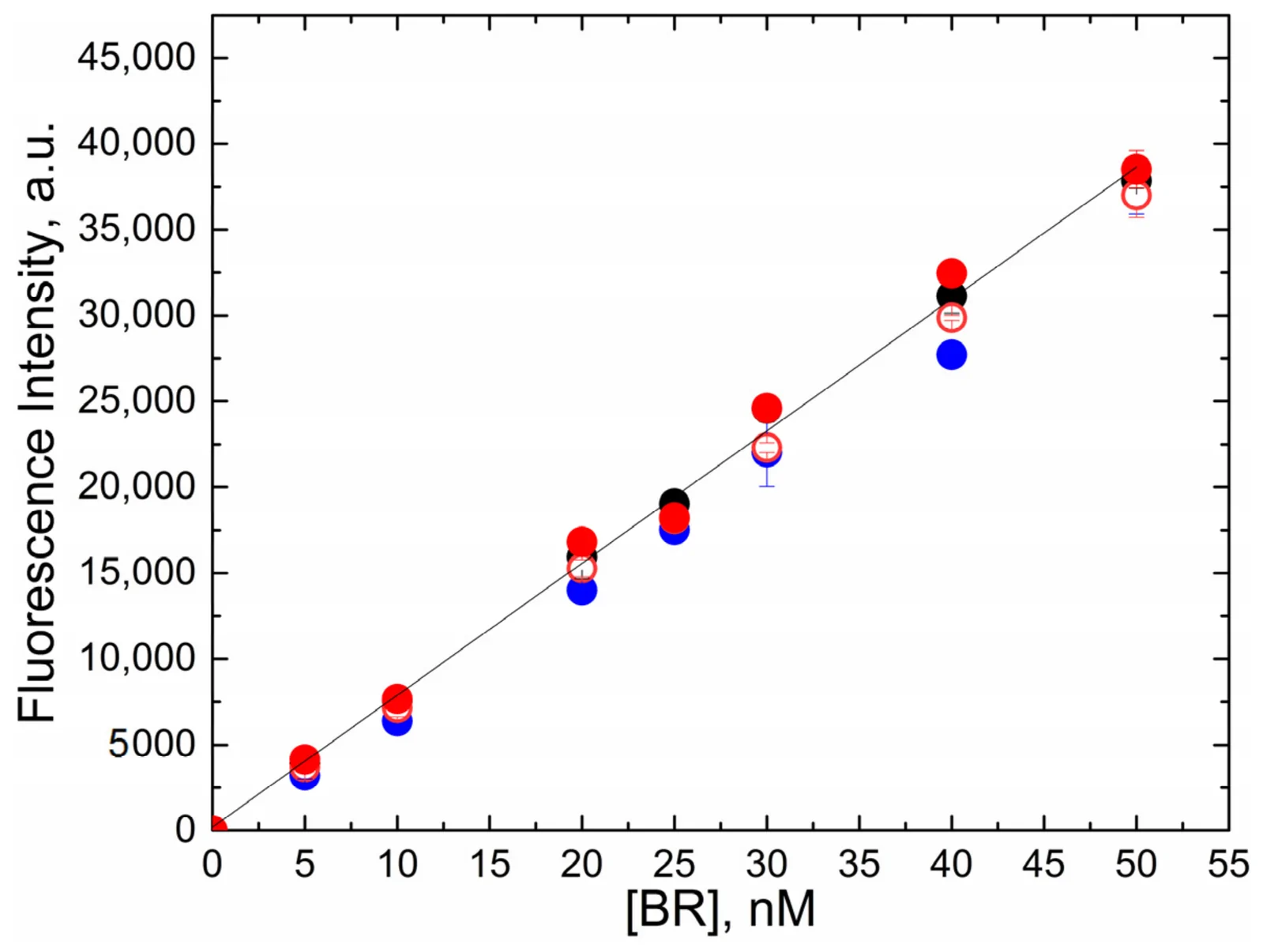
Calibration of the HUG assay. The assay was calibrated with graded concentrations of BR added to the standard solvent (4 g/L BSA in PBS) (*n* = 3, black) or rat whole blood (*n* = 2, red), rat plasma (*n* = 2, empty red), and fish plasma (*n* = 2, blue), as specified in the Methods. The background fluorescence intensities of rat whole blood (5114 ± 121), rat plasma (5271 ± 625) and fish plasma (2555 ± 166), respectively, were subtracted from the data obtained at each BR concentration. Fluorescence intensity data are shown as means ± SE. Data obtained with the standard solvent were fitted to the equation y = mx + q. The parameters obtained with the standard solvent are m = 769, q = 198, R^2^ = 0.992. The figure shows only the fitted line of the curve obtained with the standard solvent, for clarity.

**Figure 2:**
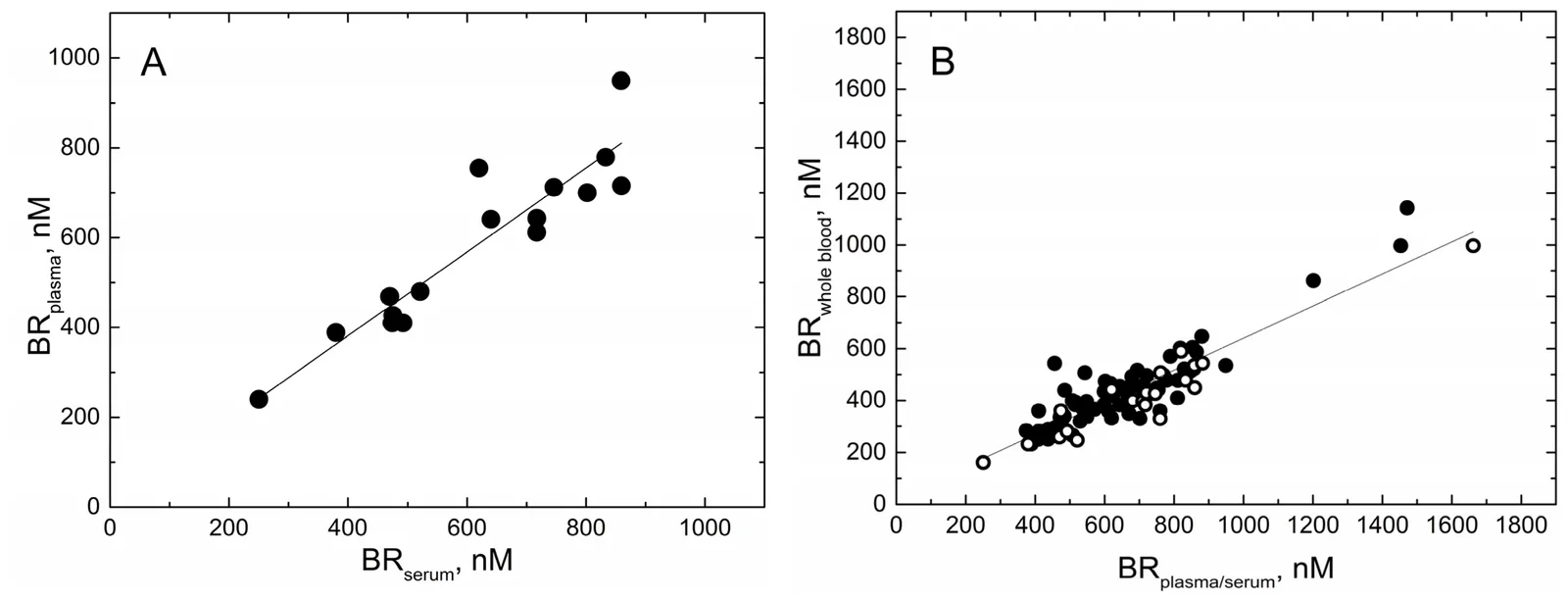
Correlation of bilirubin levels in (**A**) rat plasma vs. serum, *n* =16 (Pearson’s r = 0.927; *p* < 0.0001) and (**B**) whole blood vs. either plasma (solid black, *n* = 75) and serum (empty black, *n* = 20), *n* = 95 (Pearson’s r = 0.895; *p* < 0.0001).

**Figure 3:**
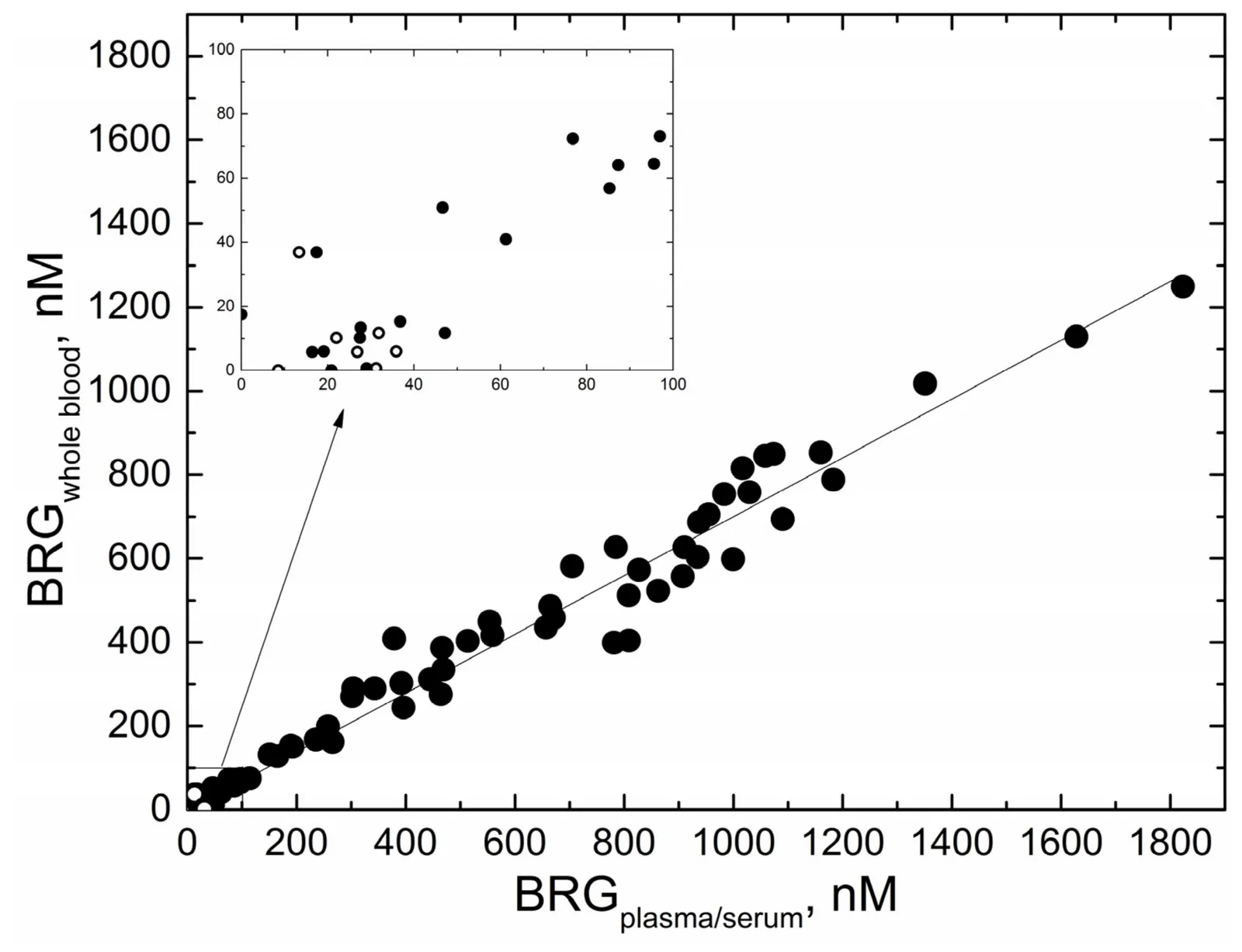
Correlation of BRG levels between rat whole blood and plasma (solid black; *n* = 65)/serum (empty black; *n* = 7) (*n* = 72). Data correlation was analyzed by Pearson’s equation (r = 0.986; *p* < 0.0001).

**Figure 4:**
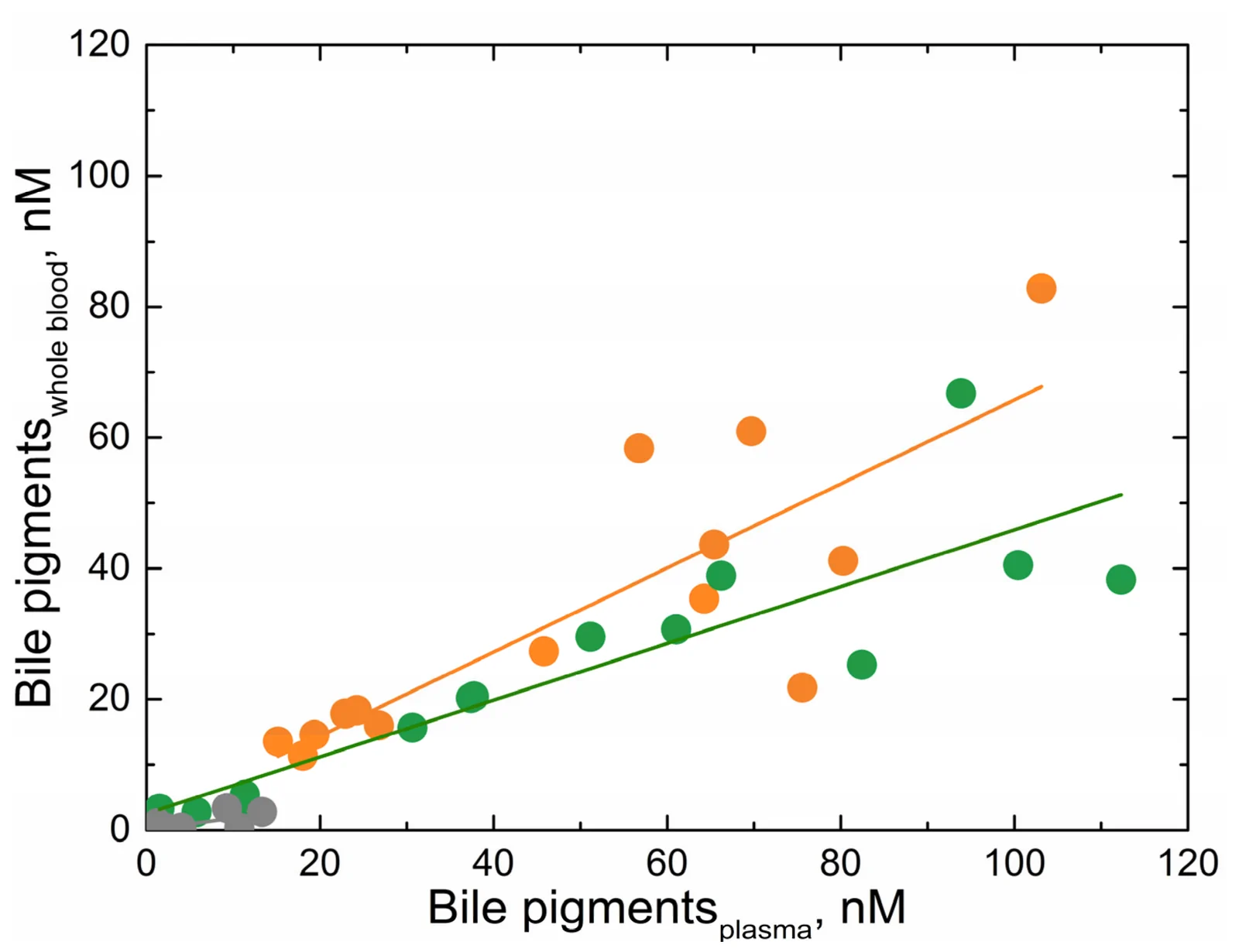
Correlation of bile pigments between whole blood and plasma of fish (*n* = 14). Data correlation was analyzed by Pearson’s equation, and the parameters were r = 0.833; *p* = 0.0002, r = 0.741; *p* = 0.0024, and r = 0.873; *p* < 0.0001 for BR (orange), BRG (gray) and BV (green), respectively.

**Table 1:** Analytical recovery of BR in the rat blood (mean ± SD, *n* = 3).

Sample	Spike Level nM	Expected BSA nM	Observed nM	Recovery %	*p*-Value
Whole Blood (*n* = 3)	Low	10.7 (±1.0)	10.4 (±2.4)	97	0.761
	Med	25.4 (±0.5)	24.7 (±2.6)	97	0.635
	High	49.8 (±5.4)	48.7 (±4.2)	98	0.913
Plasma (*n* = 3)	Low	10.6 (±1.2)	10.8 (±0.6)	102	0.801
	Med	24.5 (±1.3)	23.4 (±2.2)	95	0.508
	High	49.3 (±2.5)	47.8 (±1.3)	97	0.43
Serum (*n* = 3)	Low	10.6 (±1.2)	9.8 (±2.1)	92	0.608
	Med	24.5 (±1.3)	23.3 (±1.9)	95	0.397
	High	49.3 (±2.5)	49.4 (±2.7)	100	0.496

## Data Availability

Data is contained within the article.

## Abbreviations

The following abbreviations are used in this manuscript:

BR	Bilirubin
BRG	Bilirubin glucuronide
BV	Biliverdin
BVR	Biliverdin reductase
HUG	HELP-UnaG
PBS	Phosphate-buffered saline solution pH 7.4
BSA	Bovine Serum Albumin fraction V
